# A population based study of drowning in Canada

**DOI:** 10.1186/s12889-016-3221-8

**Published:** 2016-07-13

**Authors:** Tessa Clemens, Hala Tamim, Michael Rotondi, Alison K. Macpherson

**Affiliations:** School of Kinesiology & Health Science, York University, 4700 Keele Street, Toronto, ON M3J 1P3 Canada

**Keywords:** Epidemiology, Drowning, Age factors

## Abstract

**Background:**

Although water-related fatality rates have changed over time, the epidemiology of drowning in Canada has not recently been examined. In spite of the evidence supporting varying drowning death rates by age, information on how characteristics of drowning incidents differ by age group remains limited. The primary objective of this study was to examine the epidemiology of drowning in Canada. A secondary objective was to describe the characteristics of these drowning incidents as they vary by age group.

**Methods:**

A retrospective descriptive analysis was conducted using data that were collected for incidents occurring in Canada between January 1, 2008 and December 31, 2012. The main outcome variable was a water-related fatality, in the majority of cases (94 %) the primary cause of death was drowning. Age specific frequencies, proportions and rates per 100,000 population were calculated and compared among six age groups.

**Results:**

There were 2392 unintentional water-related fatalities identified in Canada between 2008 and 2012. Death rates (per 100,000) varied by age group 0–4 (1.05), 5–14 (0.57), 15–19 (1.27), 20–34 (1.70), 35–64 (1.44), 65+ (1.74). The male to female ratio was 5:1. Differences in the characteristics of drowning by age group were identified across: sex, body of water, urban versus rural location, time of year, activity type, purpose of activity, alcohol involvement, personal flotation device use, accompaniment, and whether a rescue was attempted.

**Conclusions:**

The study results suggest that there may be a need for drowning prevention strategies that are tailored to specific age groups. Rural areas in Canada may also benefit from targeted drowning prevention.

## Background

Drowning is a significant cause of mortality in Canada. It is the second leading cause of injury related death in children under the age of 10 and the third leading cause of unintentional injury death among Canadians under 60 [1]. Approximately 500 water-related fatalities occur in Canada each year. The drowning death rate in Canada has changed dramatically over time from over 2.0 per 100,000 in the early 1990s to 1.4 per 100,000 in recent years [2]. No formal analysis has been conducted to determine whether this change is accompanied by a change in the epidemiological characteristics of drowning in Canada.

It is well known that drowning rates vary with age [3–10]. Despite this, the manner in which the characteristics of a drowning incident vary among different age groups has not been well described.

A report that includes the epidemiology of drowning in Canada published 15 years ago indicates that toddlers (1–4) and youth (15–19) are at higher risk for drowning than the rest of the population [11], however no details on the characteristics of drowning incidents in these age groups is provided. One study conducted in the United States described the variation in drowning circumstances by age group, demonstrating that the characteristics of drowning vary greatly by age and that different prevention strategies may be needed for different age groups [3]. The study was limited by a small study region as only drownings that occurred in three counties in Western Washington state were included (*n* = 709); limiting generalizability to other jurisdictions.

Primary and secondary prevention strategies are critical to reducing mortality associated with drowning because tertiary treatment measures such as in-hospital care do not substantially affect the outcome of drowning injuries [12]. Understanding the characteristics of drowning incidents is a necessary step in designing effective drowning prevention strategies. As such, the primary objective of this study was to re-examine the epidemiology of drowning in Canada. A secondary objective was to describe the characteristics of drowning incidents as they vary by age group.

## Methods

### Study population and design

This retrospective data analysis was conducted using data collected in all provinces and territories of Canada on water-related fatalities that occurred between January 1, 2008 and December 31, 2012 by the Drowning Prevention Research Centre. All identified unintentional water-related fatalities where the incident date fell within the study period were included. The Drowning Prevention Research Centre did not collect data on cases where the death was classified as a suicide or homicide. Thus intentional drowning cases were excluded. Intent is determined based on the coroner or medical examiner’s classification.

### Data collection

Trained data collectors entered the provincial and territorial coroner’s offices annually to conduct structured reviews of the coroner’s files for all water-related deaths. Typical data sources found in the files included coroner’s investigation statement, post mortem examination report, police report, hospital records, and death certificate. A structured questionnaire was used to obtain data on cause of death; activity type and purpose of activity; and personal, equipment and environmental risk factors. The face validity of the questionnaire has been assessed several times since it was created in 1991 and questions have been altered, added or removed to ensure the most reliable and comprehensive data on water-related fatalities is collected. The Drowning Prevention Research Centre undertook an in-depth review of the questionnaire in 2010, and the revised questionnaire that was confirmed as a result of that review was used to collect the data for this study.

Project managers supervised the data collectors in each province and territory and all completed questionnaires were verified for admissibility, completeness, and internal consistency by a national project manager. Questions about the admissibility of cases were forwarded to a consultant epidemiologist for review. Data entry was done at the national level and quality controlled through double entry.

### Measures

The primary outcome measure in this study was any unintentional water-related fatality that occurred in Canadian waters. In the majority of cases (94 %) the primary cause of death was drowning. However, deaths due to hypothermia and blunt force trauma where the external cause of the death was directly related to being in or on the water were also included.

To describe the epidemiology of drowning in Canada, all variables available from the Drowning Prevention Research Centre database that are related to person, place and time were selected. These included age categorized as 0–4, 5–14, 15–19, 20–34, 35–64 and 65+; sex; body of water defined as ocean, lake or pond, flowing water, bathtub, pool, or other; urban versus rural setting based on the statistics Canada definition [13].

Additional variables were included to determine the variation in the characteristics of drowning incidents by age group. These included type of activity which describes what the victim was doing at the time of the incident, including whether or not the victim intended to be in or on the water; purpose of activity which describes whether the activity was recreational, daily living, occupational or an attempted rescue; alcohol involvement measured by blood alcohol reading collected post mortem; personal flotation device (PFD) use which describes whether or not a PFD such as a lifejacket was worn at the time of the incident in relevant situations such as boating; accompaniment which measures whether the victim was alone or with others at the time of the incident; and rescue attempt which identifies whether or not an acute rescue was attempted at a time when the victim was potentially survivable.

### Statistical analysis

Descriptive statistics were reported to summarize the characteristics of recent drowning in Canada. Mortality rates per 100,000 population per year were calculated. Denominators for rates were yearly estimates of the national population by sex and age in five year groups. Chi-square tests were performed to examine the relationship between each age group and sex, type of body of water, whether the incident occurred in an urban or rural location, time of year of incident, type of activity, purpose of activity, alcohol involvement, PFD use, whether or not the individual was accompanied at the time of the incident, and whether or not a rescue was attempted. In the event of a cell count of less than five individuals, a Fisher’s Exact Test was used. Mortality rates per 100,000 population per year were then calculated within each age group. All analysis was carried out using the Statistical Package for Social Sciences (SPSS) version 23.

## Results

During the study period, 2392 cases of unintentional water-related fatality in Canada were identified. Data on age was missing for one victim; this victim was excluded from analysis leaving 2391 water-related fatalities. Table 1 shows the frequencies and percentages within age group for each variable. Significant differences were noted across all variables (*p* < 0.05). Table 2 shows the rate of water-related fatality per 100,000 population within age group for each variable.

**Table 1:** Characteristics of unintentional water-related fatalities in Canada, 2008–2012, by age group

Characteristic^a^	Age group, No. (%)^b^							Significance
	All ages	0–4	5–14	15–19	20–34	35–64	65+	
Total	2391(100)	98(4.1)	107(4.5)	142(5.9)	592(24.8)	1033(43.2)	419(17.5)	
Sex								*P* < 0.001
Male	1967(82.3)	69(70.4)	82(76.6)	117(82.4)	519(87.7)	854(82.7)	326(77.8)	
Female	424(17.7)	29(29.6)	25(23.4)	25(17.6)	73(12.3)	179(17.3)	93(22.2)	
Body of water								*P* < 0.001
Ocean	229(9.6)	1(1.0)	2(1.9)	5(3.5)	41(6.9)	152(14.7)	28(6.7)	
Lake or pond	923(38.6)	22(22.4)	40(37.4)	71(50.0)	229(38.7)	396(38.3)	165(39.4)	
Flowing water	654(27.4)	8(8.2)	31(29.0)	51(35.9)	218(36.8)	267(25.8)	79(18.9)	
Bathtub	213(8.9)	19(19.4)	5(4.7)	4(2.8)	32(5.4)	85(8.2)	68(16.2)	
Pool	159(6.6)	41(41.8)	21(19.6)	3(2.1)	16(2.7)	37(3.6)	41(9.8)	
Other	213(8.9)	7(7.1)	8(7.5)	8(5.6)	56(9.5)	96(9.3)	38(9.1)	
Urban vs rural								*P* < 0.05
Urban	1413(59.1)	70(71.4)	68(63.6)	84(59.2)	331(55.9)	589(57.0)	271(64.7)	
Rural	970(40.6)	28(28.6)	39(36.4)	58(40.8)	258(43.6)	439(42.5)	148(35.3)	
Time of year								*P* < 0.001
Summer	1364(57.0)	64(65.3)	82(76.6)	96(67.6)	364(61.5)	544(52.7)	214(51.1)	
Rest of year	975(40.8)	34(34.7)	25(23.4)	42(29.6)	221(37.3)	456(44.1)	197(47.0)	
Type of activity								*P* < 0.001
Aquatic	594(24.8)	8(8.2)	58(54.2)	49(34.5)	185(29.0)	213(20.6)	81(19.3)	
Boating	633(26.5)	0	11(10.3)	46(32.4)	165(24.7)	308(29.8)	103(24.6)	
Transportation	378(15.8)	1(1.0)	12(11.2)	21(14.8)	110(22.9)	185(17.9)	49(11.7)	
Non-aquatic	489(20.5)	71(72.4)	20(18.7)	17(12.0)	78(12.2)	197(19.1)	106(25.3)	
Bathing	208(8.7)	18(18.4)	5(4.7)	4(2.8)	31(6.0)	81(7.8)	69(16.5)	
Purpose of activity								*P* < 0.001
Recreational	1476(61.7)	77(78.6)	92(86.0)	109(76.8)	380(64.2)	580(56.1)	238(56.8)	
Occupational	125(5.2)	0	0	0	30(5.1)	83(8.0)	12(2.9)	
Daily living	603(25.2)	21(21.4)	14(13.1)	26(18.3)	136(23.0)	265(25.7)	141(33.7)	
Rescue attempt	40(1.7)	0	0	0	13(2.2)	24 (2.3)	3(0.7)	
Alcohol involvement								*P* < 0.001
Alcohol	889(37.2)	0	2(1.9)	58(40.8)	304(51.4)	437(42.3)	88(21.0)	
No alcohol	1198(50.1)	91(92.9)	93(86.9)	70(49.3)	231(39.0)	474(45.9)	239(57.0)	
Unknown	304(12.7)	7(7.1)	12(11.2)	14 (9.9)	57(9.6)	122(11.8)	92(22.0)	
PFD while boating								*P* < 0.01
PFD worn	121(16.4)	0	6(46.2)	4(8.0)	21(11.1)	72(19.2)	18(16.2)	
PFD not worn	485(65.6)	0	7(53.8)	43(86.0)	130(68.4)	231(61.6)	74(66.7)	
Unknown	133(18.0)	0	0	3(6.0)	39(20.5)	72(19.2)	19(17.1)	
Accompaniment								*P* < 0.001
Alone	1086(45.4)	57(58.2)	14(13.1)	39(27.5)	174(29.4)	511(49.5)	291(69.5)	
With others	1200(50.2)	37(17.9)	71(66.4)	98(69.0)	401(67.7)	473(45.8)	120(28.6)	
Rescue attempt								*P* < 0.001
Attempted	1229(51.4)	93(94.9)	85(79.4)	69(48.6)	290(49.0)	496(48.0)	196(46.8)	
Not attempted	1075(45.0)	5(5.1)	21(19.6)	67(47.2)	267(45.1)	505(48.9)	210(50.1)	

^a^Some data missing for most row categories. Categories with more than 10 % missing data were: alcohol involvement (12.7 %) and PFD use while boating (18 %)

^b^All percentages are column percent, column percentages do not add up to 100 % due to missing data

**Table 2:** Rate of unintentional water-related fatality per 100,000 population per year, in Canada, 2008-2012

Characteristic^a^	Rate of water-related fatality per 100 000/year						
	All Ages	0–4	5–14	15–19	20–34	35–64	65+
Total	1.41	1.05	0.57	1.27	1.70	1.44	1.74
Sex							
Male	2.34	1.45	0.85	2.04	2.96	2.38	3.05
Female	0.49	0.64	0.27	0.46	0.42	0.50	0.69
Body of water							
Ocean	0.13	0.01	0.01	0.04	0.12	0.21	0.12
Lake or pond	0.54	0.24	0.21	0.63	0.66	0.55	0.68
Flowing water	0.38	0.09	0.16	0.45	0.63	0.37	0.33
Bathtub	0.13	0.20	0.03	0.04	0.09	0.12	0.28
Pool	0.09	0.44	0.11	0.03	0.05	0.05	0.17
Other	0.13	0.08	0.04	0.07	0.16	0.13	0.16
Urban vs rural							
Urban	0.83	0.75	0.36	0.75	0.95	0.82	1.12
Rural	0.57	0.30	0.21	0.52	0.74	0.61	0.61
Time of year							
Summer	0.80	0.69	0.44	0.86	1.05	0.76	0.89
Rest of Year	0.57	0.36	0.13	0.37	0.64	0.64	0.82
Type of activity							
Aquatic	0.35	0.09	0.31	0.44	0.53	0.30	0.34
Boating	0.37	0	0.06	0.41	0.47	0.43	0.43
Transportation	0.22	0.01	0.06	0.19	0.32	0.26	0.20
Non-aquatic	0.29	0.76	0.11	0.15	0.22	0.27	0.44
Bathing	0.12	0.19	0.03	0.04	0.09	0.11	0.29
Purpose of activity							
Recreational	0.87	0.83	0.49	0.97	1.09	0.81	0.99
Occupational	0.07	0	0	0	0.09	0.12	0.05
Daily living	0.35	0.23	0.07	0.23	0.39	0.37	0.58
Rescue attempt	0.02	0	0	0	0.04	0.03	0.01
Alcohol involvement							
Alcohol	0.52	0	0.01	0.52	0.87	0.61	0.37
No alcohol	0.70	0.98	0.49	0.62	0.66	0.66	0.99
PFD use while boating							
PFD worn	0.07	0	0.03	0.04	0.06	0.10	0.07
PFD not worn	0.29	0	0.04	0.38	0.37	0.32	0.31
Accompaniment							
Alone	0.64	0.61	0.07	0.35	0.50	0.71	1.21
With others	0.71	0.40	0.38	0.87	1.15	0.66	0.50
Rescue attempt							
Attempted	0.72	1.00	0.45	0.62	0.83	0.71	0.81
Not attempted	0.63	0.05	0.11	0.60	0.77	0.66	0.87

^a^Some data missing for most row categories. Categories with more than 10 % missing data were: alcohol involvement (12.7 %) and PFD use while boating (18 %)

By age, the highest water-related fatality rates were found among adults 65 and older (1.74/100,000), 20 to 34 years of age (1.70/100,000), and 35 to 64 years of age (1.44/100,000). Lowest rates were among those 5 to 14 years old (0.57/100,000). The proportion of male drownings (82 %) was far greater than that of females (18 %). Males had a water-related fatality rate of 2.34 per 100,000 compared to 0.49 for females. The male to female rate ratio was highest among 20–34 year olds (7:1).

Over three quarters (75.5 %) of drowning fatalities in Canada occurred in natural bodies of water with the highest proportion occurring in lakes (38.6 %) followed by flowing water such as rivers and streams (27.4 %). Teenagers 15–19 years old had the highest proportion of deaths in natural bodies of water (89.4 %), and their highest fatality rates in lakes and ponds (0.63/100,000). The most common man-made setting for drowning in Canada was bathtubs (8.9 %), followed by private pools (5.6 %). Just over half (57.7 %) of all drownings occurred in an urban environment. Children 0–4 years of age in particular had a high proportion of urban fatalities (70.6 %) and experienced their highest fatality rate in private pools (0.44/100,000).

The majority (55.7 %) of water-related fatalities in Canada occurred during the warmer months, May through August. Individuals 5–14 years of age had the highest proportion of summertime drownings (76.6 %) and 20–24 year olds had the highest summertime drowning rate (1.05/100,000).

By type of activity, the greatest proportion of water-related fatalities in Canada occurred as the result of a boating incident (26.5 %). Boating death rates were highest among 20–34 year olds (0.47/100,000). The majority (82.2 %) of boating fatalities occurred during a recreational activity. Of these, the most common activities were boating for pleasure and fishing (30.7 % and 29.2 % respectively). Fewer boating deaths occurred during an occupational activity such as commercial fishing, or a daily living activity such as fishing for food (8.7 % and 6.8 % respectively). Nine out of ten (92.0 %) boating deaths occurred among males.

For 5–14 year olds and 15–19 year olds, drowning during an in-water activity was more common (54.2 and 34.5 % respectively). Among children under the age of 5, death rates were highest for non-aquatic incidents resulting in an unintentional fall into water (0.76/100,000). Young adults 20–34 had a higher proportion of, and death rate for transportation related drowning caused by land, ice or air transportation, than any other age group (22.9 %, 0.32/100,000).

By purpose of activity, the majority of drowning deaths were recreational (61.7 %). Younger victims under the age of 5 and between 5 and 14 years old had the highest proportions of recreational drowning (78.6 % and 86 % respectively), and recreational drowning rates were highest among 20–34 year olds (1.09/100,000).

Alcohol consumption was detected in 37.2 % of the sample. Half (51.4 %) of all victims aged 20–34 had consumed alcohol prior to the incident. The next greatest proportions of alcohol use were found among 35–64 year olds and 15–19 year olds (42.3 and 40.8 %, respectively). Two thirds of adults 65 and older had one or more accompanying chronic condition (67.3 %). The most common accompanying disease found in adults 65 and older was heart disease (47.5 %). Of all victims who drowned as a result of a boating incident, two thirds (65.6 %) were not wearing a PFD. PFD use was the lowest among 15–19 year olds (86 % not worn).

Over half (51.4 %) of all victims were accompanied at the time of the incident. The age group with the highest proportion of accompaniment was 15–19 year olds (69 %). It was more common for individuals at the extremes of age to be alone at the time of the incident; 58.2 % of those 0–4 years of age and 69.5 % of those 65 and older were unaccompanied. An acute rescue was attempted in half (51.4 %) of water-related fatalities. The proportion of incidents in which an unsuccessful rescue was attempted decreased with victim age; an acute rescue was attempted for 94.9 % of 0–4 year olds and 79.4 % of 5–14 year olds, however less than 50 % of individuals 15 and older had a rescue attempted.

## Discussion

In Canada between 2008 and 2012, the highest water-related fatality rates were found among those 20 years of age and older. The vast majority of drowning victims were male. Most drowning deaths occurred in natural bodies of water, especially lakes and ponds, and over half of all incidents occurred in urban environments. More drowning fatalities occurred during the warmer months (May through August).

Comparing these results to those published in previous years [1, 11, 14] it appears that drowning deaths in Canada are down substantially among children under the age of 5; there is an 8 % decrease from the previous five year period and a 39 % decrease from the late 1990s. Drowning rates among adults, however, have either remained consistent with those previously reported or increased in the current data. The greatest increase in death rate (13 %) is found among older adults 65 years of age and older. The current age profile of drowning victims in Canada is also different from what has been reported for the global population, including other high income countries with similar drowning death rates. According to the Global report on drowning, peak drowning rates are found among children 1–4 years of age, followed by children 5–9 years of age [15]. Similarly, a study of Washington state drowning deaths published in 2003 reported the highest rates to be among 0–4 year olds, followed by 15–19 year olds [3]. In Isfahan province, Iran, the highest rates were found in age groups 15–24, 5–14, and children under 5 [8].

The substantial decline in drowning fatalities in the 0–4 age group experienced in Canada since the late 1990s could be the result of drowning prevention efforts. Such interventions include the Within Arms’ Reach campaign, which provides parents with specific and concrete parameters for their supervisory role when children under 5 are around water [16], and the efforts of numerous prevention groups to discourage the use of baby bath seats, a product that has been linked to a number of infant drownings [17–19]. The increase in the water-related fatality rate for adults indicates that more prevention strategies targeting these age groups may be warranted.

There have been no substantial changes in the male to female ratio of recent drowning victims in Canada, or the body of water where the majority of drowning deaths occur [1]. More water-related fatalities appear to be occurring in rural environments than in previous years [1]. In the current data, drowning incidents were almost equally likely to occur in an urban or rural environment. However, proportions reported previously indicated that almost three quarters of water-related fatalities occurred in urban environments. This difference cannot be accounted for by a change in where Canadians are living. In fact the proportion of Canadians living in an urban area has increased steadily overtime to 81 % in 2011 [20]. A study of drowning deaths in Ontario from 2004–2008 found that rural residence was associated with an increased risk of drowning for both males and females [21]. Increased drowning prevention strategies targeting rural areas and their residents may be warranted.

The characteristics of drowning incidents varied by age group. General patterns that emerged within each group were:

Young children 0–4 years old had a high proportion of fatalities related to falling into a pool or drowning while bathing. They were much more likely to be alone at the time of the incident than other age groups, yet almost always had an unsuccessful acute rescue attempted.

Older children aged 5–14 were at the lowest risk for drowning. They had a high proportion of pool drowning deaths and were most likely to drown in the summertime, while engaged in a recreational activity, most commonly swimming. Older children were more likely to wear a PFD while boating than any other age group.

Teenagers aged 15–19 tended to be swimming or boating during the summer months and were often accompanied at the time of the incident. Alcohol consumption was a factor in many of the incidents and teenagers were the least likely to be wearing a PFD in boating incidents.

Young adults aged 20–34 had the second highest water-related fatality rate of all age groups and the highest proportion of male victims. They tended to drown in natural bodies of water during a recreational aquatic activity or boating incident and over 50 % had consumed alcohol. There were more transportation related drowning deaths among this age group and a low proportion of PFD use while boating.

Middle aged adults 35–64 tended to be boating and had a slightly higher proportion of PFD use than some of the other age groups. Alcohol consumption was a factor in many of the incidents at this age.

Adults 65 years of age and older were more likely to drown in bathtubs and pools in urban areas and tended to be alone at the time of the incident. They were least likely to have had an acute rescue attempted. Accompanying disease, most commonly heart disease, was present in many of the decedents in this age group.

Many of the patterns in drowning characteristics observed in this study are similar to those reported by Quan and Cummings in 2003 [3]. One distinct exception is the magnitude of alcohol consumption. In their study, alcohol was a factor in 28 % of incidents in the highest risk age group as opposed to over 50 % in the present study. This difference may, in part, be due to a slightly higher prevalence of alcohol consumption (17.8 % vs 16.9 % heavy episodic drinking) and alcohol per capita consumption (10.2 vs 9.2 l of pure alcohol) in Canada than in the United States [22]. Overall, examining the characteristics of drowning incidents by age group in a national study with a much larger sample size has confirmed Quan & Cummings’ (2003) finding that characteristics of drowning incidents vary in discernable patterns by age and that different strategies may be needed for different age groups [3].

The current results suggest that in Canada, adults, especially those 65 years of age and older and those 20 to 34 years of age, should be targeted for increased drowning prevention initiatives. Additionally, programs targeting young adults should address recreational aquatic and boating activities in open water. Agencies such as Transport Canada, the Canadian Red Cross, the Canadian Safe Boating Council, and Parachute Canada stress the importance of PFD use while boating [23–26]. However the low but varying proportion of PFD use across different age groups in this study suggests that age targeted PFD interventions may be warranted, especially for teenagers and young adults.

A number of drowning prevention organisations already focus on alcohol use as a risk factor for drowning [16, 27], however messaging emphasis is typically placed on alcohol and boating. Continued anti-alcohol education should be endorsed, particularly targeting individuals 20 to 34 years of age, and campaigns should address alcohol use prior to all aquatic activities as opposed to focusing solely on boating.

While the decline in water-related fatality rates among children under the age of 5 observed in this study is encouraging, a very high proportion of the drowning fatalities among this age group occurred as a result of an unwitnessed fall into a pool. Drowning prevention agencies in Canada have long endorsed the importance of isolation fencing around backyard pools [16, 27]. Moreover, the Office of the Chief Coroner for Ontario has released recommendations that all municipalities enact isolation fencing bylaws that apply to both new and existing backyard pools [28].

The high proportion of drownings that occurred in backyard pools for children under the age of 5 in this study indicates that strategies aimed at preventing backyard pool drowning among children may require new approaches. Given that an acute rescue was attempted for almost all children under the age of 5 who drowned, one such approach may be increased rescue and resuscitation education for parents and caregivers. The outcome of paediatric drowning is closely related to the duration of submersion and the initiation of prompt and effective resuscitation [29]. As such, training parents and caregivers in effective rescue and resuscitation techniques may reduce the number of fatal drowning incidents in this age group.

### Strengths and limitations

This is the first study to assess the characteristics of drowning incidents as they differ by age group using a national population as the sample. A strength of the study is that all unintentional water-related deaths that were identified in Canada during the study period were considered. The potential for information bias caused by the proxy respondent nature of reporting on drowning deaths is a limitation of this study. Coroners and police officers often have to rely on witness statements to complete their reports. However, the data verification process undertaken by the Drowning Prevention Research Centre Canada helps to detect potential errors that are corrected by contacting the source, such as the coroner’s office or the police. Another limitation of this study is that some data were missing. Additionally, multivariate analysis is needed to confirm the identified differences in characteristics by age group and evaluate confounding bias.

## Conclusion

The recent change in the Canadian water-related fatality rate has been accompanied by a change in the age profile of victims. Additionally, more drownings are occurring in rural environments than in previous years. There are no major changes in the sex of drowning victims, body of water, or time of year when drownings occur in Canada. Characteristics of drowning incidents vary greatly by age. Acknowledging these differences may thus be useful for the design of more effective drowning prevention strategies that target specific age groups.

## Abbreviation

PFD, personal floatation device
